# Referral systems for preterm, low birth weight, and sick newborns in Ethiopia: a qualitative assessment

**DOI:** 10.1186/s12887-020-02311-6

**Published:** 2020-08-29

**Authors:** Alula M. Teklu, James A. Litch, Alemu Tesfahun, Eskinder Wolka, Berhe Dessalegn Tuamay, Hagos Gidey, Wondimye Ashenafi Cheru, Kirsten Senturia, Wendemaghen Gezahegn, And the Every Preemie–SCALE Ethiopia Implementation Research Collaboration Group, Tedros Hailu, Solomie Jebessa, Amaha Kahsay, Kemal A. Kuti, Gillian Levine, Judith Robb-McCord, Yared Tadesse, Abraham Tariku, Abubeker Kedir Usman, Abate Yeshidinber Weldetsadik

**Affiliations:** 1 St. Paul’s Hospital Millennium Medical College, Addis Ababa, Ethiopia; 2 Global Alliance to Prevent Prematurity and Stillbirth (GAPPS), 19009 33rd Avenue W, Suite 200, Lynnwood, Seattle, WA 98036 USA; 3 Defence University, College of Health Sciences, Addis Ababa, Ethiopia; 4 Wolaita Sodo University, Wolaita Sodo, Ethiopia; 5 College of Medicine and Sciences, Adigrat University, Adigrat, Ethiopia; 6 Mekelle University, Mekelle, Ethiopia; 7 Haramaya University, College of Health and Medical Sciences, Haramaya, Ethiopia

**Keywords:** Referral and consultation, Premature infant, Premature birth, Low birth weight, Newborn, Newborn health, Ethiopia

## Abstract

**Background:**

A responsive and well-functioning newborn referral system is a cornerstone to the continuum of child health care; however, health system and client-related barriers negatively impact the referral system. Due to the complexity and multifaceted nature of newborn referral processes, studies on newborn referral systems have been limited. The objective of this study was to assess the barriers for effective functioning of the referral system for preterm, low birth weight, and sick newborns across the primary health care units in 3 contrasting regions of Ethiopia.

**Methods:**

A qualitative assessment using interviews with mothers of preterm, low birth weight, and sick newborns, interviews with facility leaders, and focus group discussions with health care providers was conducted in selected health facilities. Data were coded using an iteratively developed codebook and synthesized using thematic content analysis.

**Results:**

Gaps and barriers in the newborn referral system were identified in 3 areas: transport and referral communication; availability of, and adherence to newborn referral protocols; and family reluctance or refusal of newborn referral. Specifically, the most commonly noted barriers in both urban and rural settings were lack of ambulance, uncoordinated referral and return referral communications between providers and between facilities, unavailability or non-adherence to newborn referral protocols, family fear of the unknown, expectation of infant death despite referral, and patient costs related to referral.

**Conclusions:**

As the Ethiopian Federal Ministry of Health focuses on averting early child deaths, government investments in newborn referral systems and standardizing referral and return referral communication are urgently needed. A complimentary approach is to lessen referral overload at higher-level facilities through improvements in the scope and quality of services at lower health system tiers to provide basic and advanced newborn care.

## Background

The World Health Organization (WHO) defines referral as “a process in which a health worker at one level of the healthcare system, having insufficient resources (drugs, equipment, skills) to manage a clinical condition, seeks the assistance of a better or differently resourced facility at the same or higher level to assist in, or take over the management of, the client’s case” [1]. An effective referral system is an essential component of the health system to improve outcomes for mothers and newborns around the time of childbirth [2]. Early illness detection requiring advanced care not available at all facility levels, coordinated with a well-functioning referral system, can significantly reduce maternal and newborn mortality and morbidity [3–7].

Decisions by initiating facilities to refer preterm, low birth weight (LBW), and sick newborns depend on factors such as severity of the disease, providers’ experience and scope of practice, availability of advanced care services, societal culture and norms, as well as socioeconomic and educational status of the family [6, 8]. When initiating a referral, communication on the reasons for referral with the receiving facility can reduce treatment delay, avoid system overload, and enable utilization of expertise at advanced clinical hubs. In circumstances when services are not available for the preterm, LBW, and sick newborn in the initiating facility, parents or other caregivers should be properly counseled on the reason for referral and receive properly documented referral papers. Communication with the receiving facility should be ensured.

The Ethiopian public health system is a 3-tier, health care delivery arrangement [9]. The first level is the primary health care unit (PHCU) comprised of health centers, each serving 15,000 to 40,000 people, and in rural areas, several satellite health posts serving a population of 3000 to 5000 each. Primary-level hospital care is provided by a primary hospital serving a population of 60,000 to 100,000 [9]. The second tier is a general hospital which is expected to serve a population of 1.0 to 1.5 million and serves as a first referral center. The third tier comprises specialized referral hospitals serving a population of 3.5 to 5.0 million [9]. These 3 levels are expected to interact through a referral system to allow exchange of information and patients. Preterm, LBW, and sick newborns are referred within this system. Public facilities are expected to provide free ambulance services for referrals between facilities.

Health care services in Ethiopia are delivered through extensive national programs and networks in which the referral system is one area of focus [10]. Referrals begin at lower tiers of the primary health care system and continue to higher ones although there can be horizontal referrals between similar-level facilities at the request of patients [11]. Referral system implementation, however, has been facing challenges as a result of resource- and management-related constraints [12].

With a population of over 100 million, Ethiopia is the second most populous country in Africa. Close to 78% of the population live in rural areas. Over the last two decades, it has achieved remarkable increases in the coverage of essential health services, particularly in the areas of maternal, newborn, child and nutrition-related healthcare. Between 2005 and 2019, coverage of 4 visits of antenatal care, health facility delivery and vaccination with all basic child vaccines increased from 12 to 43%, 5 to 48%, and 20 to 43%, respectively. Similarly, treatment-seeking for childhood illnesses and the overall use of modern health services increased substantially. The average annual number of health facility visits per capita, a proxy for the overall health service use rate, increased from 0.27 in 2005 to 0.9 in 2019 [13]. The health extension program began in 2003 to improve access to health services in rural and medically underserved areas, and has become an important source of maternal and newborn health services for rural communities in Ethiopia [14]. Health extension workers (HEWs) are the foundation of the health extension program. They are primarily female, and as of 2018, 42,000 workers were deployed throughout Ethiopia [15].

Prior studies have shown low effectiveness and efficiency in the maternal and newborn referral system, and thus capacity and performance improvements are recommended [12, 16]. However, no qualitative studies in the literature have identified the contextual and structural factors influencing referral conditions in Ethiopia for preterm, LBW, and sick newborns. This study used qualitative methods to assess the referral system for preterm, LBW, and sick newborns and identify barriers for its effective functioning across the primary health care unit in Ethiopia.

## Methods

### Study design

A qualitative assessment was conducted in multiple sites across 3 regions of Ethiopia (Amhara, Oromia, and Addis Ababa). Data collectors conducted (1) in-depth interviews (IDIs) with mothers of premature, LBW, or sick newborns who received care within the government health care system or delivered in the community (hereafter “mothers”); (2) IDIs with obstetric and newborn care providers and HEWs associated with study health facilities (hereafter “providers”), (3) key informant interviews (KIIs) with facility administrators in the public health care system (hereafter “facility administrators”), and (4) focus group discussions (FGDs) with providers in study health facilities. Demographic data were collected using a simple interviewer-administered questionnaire. This article conforms to the Standards for Reporting Qualitative Research [17]. The MEASURE Evaluation Project toolkit for assessing referral systems was used to develop a framework to guide our assessment of referral services for preterm, LBW, and sick newborns in Ethiopia [18].

### Study sites

The Federal Democratic Republic of Ethiopia is among the most populated countries in Africa, with a 2018 population estimate of 107 million [19]. One urban and 2 rural sites were selected to allow for assessment and descriptions of different settings and contexts in Ethiopia. Table 1 shows study region characteristics.

**Table 1:** Characteristics of Study Regions^a^

Characteristics	National	Addis Ababa	Amhara	Oromia
**Demographic Indicators**				
Proportion urban population, %^b^	16.2	100.0	12.3	12.4
Total fertility rate, No. children per woman	4.6	1.8	3.7	5.4
Proportion of women who are literate, %	42.0	87.8	44.9	37.3
Proportion of women who have a bank account, %	15.1	53.6	20.9	8.4
Proportion of women who own a mobile phone, %	27.3	87.0	21.2	23.3
Proportion of men engaged in agriculture, %	71.7	2.4	76.8	79.0
**Mortality Rates**				
Under-5 mortality, No. per 1000 live births	67	39	85	79
Infant mortality, No. per 1000 live births	48	28	67	60
Neonatal mortality, No. per 1000 live births	29	18	47	37
Low birth weight rate, %	12.7	11.5	22.2	13.1
**Maternal and Child Health Services Indicators**				
Proportion of pregnant women receiving antenatal care from a skilled provider, %	62.0	96.8	67.1	50.7
Proportion of deliveries in a health facility, %	26.0	96.6	27.1	18.8
Proportion of women with a postnatal checkup in first 2 days after birth, %	17.0	55.4	21.9	11.8

^a^ Data from the *Ethiopia Demographic and Health Survey 2016,* except as denoted in footnote b [20]

^b^ Data from the 2007 Ethiopian National Census [21]

Settings and facilities were selected in collaboration with the Federal Ministry of Health (FMOH) based on an aim to broadly represent typical contexts while considering national priorities, civil security, population size, existing health infrastructure, and existence of other ongoing research/projects that could impede implementation. A total of 65 health care facilities participated in the study (Table 2).

**Table 2:** Number of study sites and number of participants by region

	Total, No.	Amhara, No.	Oromia, No.	Addis Ababa, No.
Number of Study Sites in Each Region				
Health post	27	13	14	0
Health center	32	8	3	21
Primary hospital	2	1	1	0
Secondary hospital	2	n/a	1	1
Tertiary hospital	2	n/a	n/a	2
Completed Method Type				
In-depth interview, Mother	22	4	0^a^	18
In-depth interview, Provider	21	8	9	4
Key informant interview, Facility administrators	37	14	11	12
Focus group discussion, Provider	23^b^	9	4	10

^a^ Recently delivered mothers in Oromia could not be included in the study due to geography and limited study resources. n/a = non-applicable

^b^ Comprised 96 individuals participating in the 23 FGDs

### Sampling and recruitment

We used purposive convenience sampling for recruitment of staff and clients from a subset of health facilities at each tier of the public health care system in each region, including health post (HP), health center (HC), and primary, general and specialized hospitals [22]. Eligibility criteria of participants varied by study tool (IDI, KII, or FGD).

IDIs: Mothers who delivered small (i.e., preterm, LBW) or sick newborns at secondary and tertiary hospitals in Addis Ababa and at HPs, HCs and primary hospitals in Amhara were identified from delivery and discharge registers and recruited. If < 18 years of age, parental or guardian consent was obtained. A total of 22 mothers completed the IDI (Table 2). Participants were offered their choice of languages (Amharic or Oromiffa) for the interview. IDIs were also conducted using the FGD instrument in Amharic with 21 HEWs currently working in a study facility with 6 months experience and currently providing maternal/newborn care (Table 2).

KIIs: A subset of HPs and HCs, and all public primary, secondary and tertiary hospitals (Amhara, Oromia, and Addis Ababa, with a focus on Kirkos and Yeka sub-cities) were included. All clinical, nursing, and administrative leads at the study health facilities involved in pregnancy, labor, and delivery/obstetrics and gynecological, postnatal, and newborn care services at the facility were recruited. 37 facility administrators participated in Amharic or Oromiffa (Table 2).

FGDs: Clinical, nursing, and midwifery staff (at HCs and hospitals), and HEWs (at HPs) currently working in a study facility with 6 months experience and currently providing maternal/newborn care were included. A total of 96 providers participated in 23 FGDs (Table 2). All FGDs were conducted in Amharic.

### Data tools

A brief demographic questionnaire was created to gather basic information on age, parity, marital status, education and profession (provider and administrator only). Semi-structured guides were developed using a multistep process. First, based on the study framework, the scope of each tool was defined, and questions were drafted. After an initial training, data collectors pre-tested the instruments at St. Paul’s Hospital Millennium Medical College with interviewees similar to the target sample: 2 FGDs, 3 IDIs, and 5 KIIs. Adjustments were made for flow, content, terminology, prompts, and instructions.

IDIs: Mothers were asked 19 questions about decisions, preferences, and experiences of care during their most recent labor and delivery, postpartum, and post-discharge periods, and completed a survey of 71 closed-ended questions including demographics, pregnancy history, and details regarding their most recent pregnancy and delivery.

KIIs: Facility administrators were asked 20 questions about policies and guidelines, programs, facility preparedness, and referral transfer systems, with respect to preterm, LBW, and sick newborns, and completed a survey of 8 closed-ended questions including demographics and details regarding their current leadership positions. Participants were offered their choice of languages (Amharic or Oromiffa) for the interview.

FGDs: Providers and facility administrators were asked 36 questions about evidence-based practice, information systems, referral systems, health workforce, leadership, client experience of care, respectful care, and quality and program insights, with respect to preterm, LBW, and sick newborns, and completed a survey of 7 closed-ended questions including demographics and details regarding their current professions. Ninety-minute FGDs were separated by facility and by cadre of provider (HEWs, nurses, midwives, general practitioners, and neonatal specialists), with 4–8 participants per group. At health facilities and health posts with few providers, staff and HEWs participated in individual interviews using the FGD guide.

### Research team composition, training, and supervision

The research team was composed of investigators, data collectors, and data analysts with backgrounds in public health, medicine, and anthropology. Experienced data collectors were trained on the study objectives, participant selection, instruments, and interview skills. Supervision was conducted using a supervision checklist.

### Data collection

Data were collected from December 2017 to February 2018. Ethics approval was obtained from the Institutional Review Boards of the St. Paul’s Hospital Millennium Medical College, Addis Ababa, Ethiopia (IRB No. PM23/111), and Project Concern International (IRB No. 25). Letters of support were secured from all institutions and offices where data were collected. We obtained consent from potentially eligible and interested participants in their preferred language and informed them that their participation would be voluntary and there would be no professional or personal consequences nor benefits to participation. Mothers were given the option to read or hear their consent form according to their literacy level. To avoid possible coercion, no financial incentives were provided. Interview data were reviewed from all 3 study regions periodically during data collection until data saturation was reached (indicated by thematic repetition within subsamples).

### Data management

Demographic survey data were recorded on a tablet or log sheet, and IDIs/KIIs/FGDs were recorded digitally. For each FGD, a notetaker supplied written notes to supplement the recordings. Recordings were transcribed by experienced transcriptionists and subsequently translated by professional translators. Translated transcriptions were spot-checked for accuracy by a third team member.

Every effort was made to maintain participants’ confidentiality during data collection and manuscript preparation. All audiotapes were destroyed immediately following transcription. No names are attached to any of the data. In the results, quotes are identified only by source (i.e., IDI, KII, FGD), location (AMH = Amhara, ORO = Oromia, AA = Addis Ababa), and by facility level (HP = health post, HC = health center, HOSP = hospital) where relevant to the findings.

### Analysis

Demographic questionnaire data were analyzed using Excel. Qualitative data were entered into NVivo [23] version 12 and analyzed using thematic content analysis. The analysis team, including researchers involved in the project design and qualitative coders, developed a codebook using the following steps: initial codes derived from study goals and instrument questions; codes adapted and augmented by reading 2 transcripts and the conceptual framework; codes tested by multiple coders on 3 additional transcripts; and codebook edited as appropriate. All transcripts were open-coded using the final version of the codebook to capture key themes and relevant ideas. Each transcript was coded by 2 separate coders. Any disagreements were resolved by the lead analyst who reviewed all discrepancies and discussed them as necessary to reconcile coding. Once coding was complete, code reports were produced for each code, cleaned, and data were annotated and summarized into domains and subdomains.

## Results

### Description of sample

Mothers were from Addis Ababa (62.5%), Amhara Region (15.6%), and Oromia Region (21.9%). Among mothers, the largest proportion of respondents were 20 to 29 years old and primiparous (Table 3).

**Table 3:** Background characteristics of mothers, providers and facility administrators

	Mothers ***n*** = 32 ^a^	Providers ***n*** = 96	Facility Administrators ***n*** = 37
Characteristics	No. (%)	No. (%)	No. (%)
Age, years^b^			
15 to 19	8 (25.0)	0 (0)	0 (0)
20 to 29	18 (56.3)	78 (81.3)	24 (66.7)
30 to 39	6 (18.8)	15 (15.6)	10 (37.8)
40 and older	0 (0)	3 (3.1)	2 (5.6)
Sex			
Female	32 (100.0)	55 (57.3)	21 (56.8)
Male	n/a	41 (42.7)	16 (43.2)
Gravid			
Primiparous	19 (59.4)		
Multiparous	13 (14.6)		
Profession			
Health extension worker		0 (0)	13 (35.1)
Midwife		54 (56.3)	1 (2.7)
Nurse		29 (30.2)	15 (40.5)
Health officer		9 (9.4)	7 (18.9)
Physician		4 (4.2)	0 (0)
Neonatologist		0 (0)	1 (2.7)
Facility level			
Referral hospital		13 (13.5)	2 (5.4)
General hospital		0 (0)	3 (8.1)
Primary hospital		12 (12.5)	1 (2.7)
Health center		71 (74.0)	16 (43.2)
Health post		0 (0)	15 (40.5)
Region			
Addis Ababa	20 (62.5)	59 (61.5)	12 (32.4)
Amhara	5 (15.6)	22 (22.9)	14 (37.8)
Oromia	7 (21.9)	15 (15.6)	11 (29.7)

^a^ n = 32 for the demographic survey, *n* = 22 for completed interviews

^b^ One facility administrator participant not reported

Among providers, 61% were from Addis Ababa, 92% were ages 20 to 35, 74% were from health centers with the remaining from primary and referral hospitals, and 30% were employed as nurses (Table 3).

For facility administrators, respondents were proportionally divided across all 3 regions, 66.7% were ages 20 to 29 years, 56% were employed as midwives, and 40% were employed as nurses (Table 3).

The need for rapid transfer of preterm, LBW, and sick newborns to higher levels of care when clinical complications occurred underscored the role of the referral system in accessing services. However, the referral system failed to function effectively due to numerous logistic and systemic problems. Four major themes were related to barriers for the newborn referral process and networks: (1) transportation, (2) communication, (3) lack of, or non-adherence to, newborn referral protocol/guidelines, and (4) family refusal of newborn referrals (Table 4).

**Table 4:** Themes and sub-themes from thematic content analysis of responses

**Themes and Sub-themes**	
Transportation barriers inhibiting effective referrals	
Long distances	
Poor road networks	
Lack of appropriate transportation	
Communication barriers inhibiting effective referrals	
Phone unavailable or not answered at receiving facility	
Non-clinical contact at receiving facility	
No feedback from receiving facility	
Referral protocol and guideline failure	
Absence of protocol or guideline	
Lack of training on referral processes	
Family refusal for newborn referrals	
Fear of the unknown	
Low expectations for referral outcome	
Referral refusal due to financial constraints	

### Transportation barriers inhibiting effective referrals

Long distances, poor road networks, and lack of appropriate transportation hindered use of referral health facilities. Providers and administrators in Addis Ababa reported that referred patients coming from rural villages were delayed due to long journeys by ambulance or other means of transportation. In the case of preterm, LBW, and sick newborns, time is of the essence. Participants were concerned that delays often meant the difference between life and death. Even when a referral was not completely blocked or denied, a delay may have resulted in morbidity or mortality. Reasons for delays included distance between referring and receiving institutions, traffic jams, and ambulances arriving late after being summoned. As one participant described*,* “The problem is there are times when, even if the ambulance reached us after long hours of journey, we sometimes lost the infant [who may die] as s/he might reach the facility very delayed” (AA-FGD-HOSP).

A key informant cited problems related to road infrastructures as one challenge for effective infant referral and echoed a common complaint, “The infrastructure is challenging, the topography makes our referral linkage challenging since clients come from far villages where the topography makes it difficult to reach here” (AMH-KII-HC).

Problems of referral transportation that particularly related to absence or limited availability of ambulances were also been mentioned by FGD participants in Addis Ababa. For example,

“One of the problems in the referral system is the absence or difficulty of getting ambulance. We have big problem in ambulance at this time; when we call to the service center for ambulance they say, ‘What can we do? Just take your own solution.’ There are even ambulance drivers who warn us not to call them for the service they are supposed to render; so the big problem in referral system is an ambulance problem” (AA-FGD-HC).

A mother from Addis Ababa complained about her experience of needing an ambulance in the case of an emergency: “I thought I would get an ambulance when my baby was referred to the higher hospital. However, I couldn’t get one. So, I had to get a contract taxi which was damned expensive in order to get my baby to this hospital” (AA-FGD).

In some cases, ambulances were prioritized for adults but not for infants which resulted in parents refusing referrals. A provider from Kobo explained, “The problem is ambulance service is not given for newborn referrals in our facility. The existing ambulance only serves for women in labor, and not for other emergencies or referral services. As most of our clients are coming from the rural remote, so lack of ambulance service would make them decline to the referral of their sick babies” (AMH-FGD).

Lack of an accompanying provider, trained provider or provider with necessary oxygen or equipment all hindered safe and effective referrals for preterm, LBW, and sick newborns. As one facility administrator described: “There are some health centers that send the baby by movable ambulance; just they call to 939 to get ambulance and they send the baby by that ambulance but the driver of the ambulance does not know about the case, the ambulance does not have oxygen and there is no provider with the sick baby*”* (AA-FGD-HOSP).

When conventional ambulances were not available, parents were compelled to pay for alternative transportation. For parents without adequate funds, this scenario resulted in parents’ refusal of referral. As one health center interviewee explained, “When there is no ambulance service, you would see parents hesitate or refuse to go to the referral destination because they couldn’t afford the transportation cost. We usually ask them why they refuse to go, and when we learn that the refusal is related to finance we would try to raise some money, give it to them and arrange transport” (AMH-FGD-HC).

### Communication barriers inhibiting effective referrals

In the referral process, the initiating facility was expected to provide a referral form, communicate in advance with the receiving facility to make arrangements, and provide information to the patient or their family about the referral. The receiving facility was expected to anticipate the arrival, provide care and follow-up for the patient, and send back the referral form and feedback to the initiating facility to confirm or refute the appropriateness of referral. However, often scarcity of resources, including insufficient numbers of specialists and their high rate of turnover, as well as lack of communication technologies, contributed to ineffective referral communications between the initiating and receiving facilities.

Many of the FGD participants among the health care providers reported using advance communications between the initiating and receiving facilities’ liaison offices. However, the communication was conducted primarily through telephone conversations, and the mothers or parents of the referred infants often were not provided with referral forms. Even those facilities that used referral forms may have sent incomplete forms or no medical records with referred infants. Additionally, telephone communication may have been used only to ascertain the availability of beds at the receiving facility as the sole requirement for the referral.

“The liaison office usually communicates with the receiving facilities for availability of beds, however, personally I prefer that the communication should be with similar units, for example, to communicate our neonate unit with Black Lion Hospital neonate unit directly instead of making the communication through liaison office which only checks the availability of beds” (AA-FGD-HOSP).

Some of the participants were also concerned that telephone communication failed when the receiving telephone was not answered due to negligence, work overload, or equipment failure; “We sometimes never get the referral network at the time we need it … We solve this by calling directly to head of health bureau … we managed to get them by their personal phones and make them ready to accept our referrals” (AA-KII-HC).

In some instances where the receiving facility was far from the initiating facility, a letter would be sent to the receiving facility to initiate communication. For example, “The methods we use usually differ according to the distance of the transfer facility from our facility. If it is close by, then we communicate in person. However, if it is not then we communicate through letters” (KII-ORO-HC).

Many participants witnessed that there was no formal exchange of feedback between the initiating and the receiving facility using completed referral forms; rather, feedback was mostly conveyed during facility meetings or informal conversations. One health center interviewee described a mutable and relatively ad hoc system for conveying feedback: “The feedback system is very poor. Normally feedback has to be written to the sender unit after the sick baby came and completed the treatment in our facility. However, we are not implementing this. … If it is internal referral, they go in person and will ask how it went, how did they do it, and will take the history from there. However, we have no culture of written feedback to the sender facility in general” (KII-ORO-HC).

### Referral protocol and guideline failure

In some of the study settings, participants were unaware that referral protocols or guidelines for newborn referral services existed. Although the protocols were in existence for over 5 years prior to the study period, many of the providers reflected that they had no access to protocols, as one of the FGD participants reflected: “As to my knowledge, there is no such policy and I have not heard of it. I would know if it existed. To date there is nothing to guide us how to care and refer small and/or sick babies. I have not heard about any referral policy” (KII-AMH-HC).

Most participants in Addis Ababa knew that referral protocols or guidelines existed but indicated that they were either too busy to follow the protocol or did not think it important for their clinical decision-making, including decision for referrals: “Only one protocol is available in this hospital; most of the time the medical interns use the protocol because they need to know more. In addition, those who are not trained for neonatal intensive care, I think, they also needed it more” (FGD-AA-HOSP).

### Family refusal for newborn referrals

Decisions for referral originated with the provider or the parent. While parents sometimes chose to self-refer due to dissatisfaction with lower level facilities or lack of understanding about how the leveled system was intended to function, only providers were technically qualified to refer preterm, LBW, and sick newborns. However, once a provider initiated a referral, the decision to accept the referral was in the hands of the parent(s) who could follow through with the referral or not. Parents indicated 3 primary reasons for refusing a referral for their newborn: fear of the unknown, low expectation for survival outcome as a result of the referral, and financial constraints linked to referrals.

#### Fear of the unknown

Referral of a preterm, LBW, and sick newborn was considered an emotionally overwhelming event for most of the interviewed mothers. Mothers of preterm, LBW, and sick newborns said that they feared the unknown and assumed that their newborn was referred because of a totally unmanageable illness. One mother noted*,* “After I see that I am having a baby very small and sick, I couldn’t contain my emotion and burst with tears for the whole day. It was a difficult moment for me to see my baby’s case was even more complicated than mine. Moreover, no one has discussed me about the problem; they just only told me my baby need to be referred to another facility. I also haven’t seen providers advising other mothers about their sick newborns” (IDI-AMH).

Most of the mothers complained they had received very little or no information or counseling from the provider on the referral or condition leading to referral, and thus felt blindsided by the referral. Believing their child was destined for mortality if referred, they defaulted to familiar traditions*:* “One of the problems we face here is the community thinks patients will surely die if they are referred, and thus, they don’t like to be referred. They prefer to get some care here than to be referred as they would be in fear thinking what will happen to them far in the referral facility away from home; you also feel worried when you see them worried” (KII-AMH-HC).

Participants repeatedly described referrals as a frightening experience that drove them to remain at home to conduct trusted cultural and religious rituals to protect their infants; referral was seen as a last resort, only followed out of desperation: “Most of the time the reason for refusal of referral is that when [parents] learn that their newborn is referred, they want to go home and do cultural things, rituals, out of fear that the baby may die. Sometimes, when we were able to get their phone numbers and urge them to go to referral facility, they only get back to it after they knew that the rituals couldn’t work for them. So, these are the problems for delay for referral” (FGD-AMH-HC).

#### Low expectations for referral outcome

Mothers of sick newborns saw referrals as bad fate for infants already on the verge of death and did not expect better outcomes would result from taking preterm, LBW, and sick newborns to referral facilities. Moreover, some families preferred sick newborns die at home than somewhere far away because many Ethiopians perceive newborns are not yet full members of a family before they are baptized. There is also a common myth that small babies are destined to die. One participant explained it clearly: “We refer preterm or low birth weight babies. While doing so, one of the big challenges I observed is that the parents are not willing or happy when referred. They say, ‘If you don’t have room here we would take the newborn home and let it die there.’ They don’t value newborns” (FGD-ORO-HC).

#### Referral refusal due to financial constraints

Preterm, LBW, and sick newborn referral could be expensive when parents had to shoulder the cost of transport to the referral destination, but that was not the only expense. In most instances, relatives had to accompany the referred newborn, thus incurring additional costs for accommodation, food, and other expenses. Interviewees talked about parents selecting health facility options where they knew they could stay with, and be supported by, extended family. A provider suggested that increasing community awareness would be helpful*:* “When we refer the newborn to a particular facility, the family only wants to go to another facility where they can easily find a family member or relative in order to get support and lessen the expenses rather than saving the newborn’s life. If they couldn’t find close relatives, or if the relatives are not willing to welcome them, they would prefer to stay home” (FGD-AMH-HC).

Families of referred preterm, LBW, and sick newborns also anticipated they would be responsible for covering the cost of drugs and medical treatment in higher-level referral facilities; this discouraged them against accepting the referral: “When we refer the newborn, parents assume they will be requested more payment at the receiving hospital. They may think how to afford that payment and decide to take the sick baby home even if they accept the referral. I know there are many parents who go home after we refer them especially those from rural Oromia, because they think their economy may not afford treatment costs and to buy drugs; sometimes we inform them the payment is very minimal when we refer them” (FGD-ORO-HC).

## Discussion

This qualitative study assessed Ethiopia’s public referral system that supports the facility care of preterm, LBW, and sick newborns. Ethiopia’s health system has been in transition with notable improvements. Institutional deliveries have increased from 5% in 2000 to 10% in 2011, and 26% in 2016 [19]. However, only 17% of women and 13% of newborns received a postnatal check within the first 2 days of birth [19]. This study reports persistent challenges in the referral system in Ethiopia. Barriers in communication, transportation related challenges, poor adherence to protocol, fear of unknown outcomes and resource-constraints have limited its effective execution.

Functional referral systems offer multiple benefits for patients to: avoid unnecessary costs, receive appropriate and timely care, avoid unnecessary resource wastage, avoid potential barriers to access to care, and facilitate communications among health care providers [24]. Functional referral monitoring systems will allow decision-makers to track how often referrals are being made to different facilities and services, the types of services for which clients are most often referred, whether clients are able to take advantage of the referrals, and whether adequate follow-up is provided after the fact [25, 26].

Participants from all groups across all the study settings reported that transportation to receiving facilities was a significant barrier due to a widespread dearth of ambulance services for the referral of preterm, LBW, and sick newborns. In rural areas, existing ambulance services were often reserved only for transportation of laboring mothers and other emergency cases, rather than for newborn referrals. An ambulance scarcity was also found in studies in rural southern Ethiopia [27], Sierra Leone [28], and Ghana [24], as well as in many countries of the developing world in general [29].

In urban Addis Ababa, participants noted that the few available ambulances were not functioning well due to lack of proper communication between ambulance service centers and initiating facilities when service centers did not respond to phone calls and ambulance drivers were reluctant. Findings from a study by Austin et al. in Addis Ababa revealed similar communication problems during emergency obstetric care and referrals [12]. Addis Ababa was over-represented in our sample due to much higher number of births and research resource issues that limited the period of study, and yet our findings show that even in Addis Ababa, extensive referral problems are prevalent. This suggests that in more rural areas of the country, the referral challenges are likely to be even more pronounced.

Lack of availability of, or access to, government-supported ambulance services for preterm, LBW, and sick newborns in both rural and urban settings points to the need for the FMOH to adequately finance and support a functional referral system as an important component of quality newborn care. Improving the referral system would also contribute to increasing the Ethiopian health system’s readiness to provide quality care to newborns [30]. This would directly support the Ministry’s national strategy for newborn and child survival for 2016–2020 target to reduce child morbidity and mortality through integrated child health care provisions and functional referral services [31]. The national protocol for newborn care and referral was not being effectively implemented in the health care system. With strictly implemented government referral protocols, the relevant referral services would have been available, and resource gaps would have been filled.

With the persistent challenges to the availability of and access to quality advance care and ambulance services, maternal in-utero transfer to centralized perinatal care could be an effective measure to improving neonatal outcome especially for high-risk pregnancies and threatened preterm birth. Maternal in-utero transfer is not called out as one of 34 cost-effective evidence based interventions in the National Strategy for Newborn and Child Survival [31]. However this approach is implied in the 3-tiered health system that calls for appropriate and timely referrals [9].

Uncoordinated pre-referral communication between initiating and receiving facilities was repeatedly reported by participants across the study settings. Communication was characterized by informal exchange of information by telephone only to confirm bed availability and briefly convey the patients’ condition upon referral acceptance. Study participants shared that although referral forms were available in some facilities, health care providers seldom completed or used forms because they were perceived as irrelevant and time consuming; instead providers would be rushed to call receiving facilities and send newborns after referral acceptances were confirmed. The same was true for written feedback from receiving facilities to initiating facilities; phone calls were sometimes made if the receiving facility was unclear about the patient’s condition, but otherwise the receiving facility often did not communicate at all. Without routine documentation, referral system monitoring is very challenging. Ineffective referral communications and resulting negative effects have also been reported in qualitative studies conducted in Ghana [24], Brazil [32], and Guatemala [33]. Improvements to monitoring and evaluation are critical to ongoing strengthening of the overall referral system.

Referral refusal is commonplace for various reasons, including geographic and financial constraints, illness severity, and long wait time. When families refuse referrals, reasons should be sorted out and addressed accordingly, and appropriate options should be offered [4]. Our findings revealed that the fear of high costs for services including transportation and drugs as well as other informal payments for accommodations made families reluctant to accept referrals. This finding confirmed a qualitative study from southern Tanzania where mothers of sick newborns complained about high treatment costs at referral centers and transportation expenses [34]. Conversely, referral-related expenses were shown not to affect acceptance in a study conducted in South Africa [6]; however, the setting was a more developed urban periphery, unlike the urban-rural mix of our study settings.

Some rural families in this study reportedly compromised their newborns’ well-being, preferring to allow infants to die at home rather than be referred because newborns were not perceived to be full members of the household. This is consistent with studies from central Ethiopia [8] and rural Uganda [35] in which delays in decision-making and care-seeking for sick newborns were due to families’ perceptions that newborns’ health was not the families’ highest priority.

### Limitations

There are limitations to this study and analysis. Although study sites were drawn from 3 different regions of the country, the relative number of recruitment locations within these regions were few and not necessarily representative of the entire country. Similarly, mothers, providers and facility administrators who were interviewed may not be representative of all patients and clinicians. Mothers in Oromia were not available for IDIs due to geography and difficulty in contacting them given our resources.

## Conclusions

Access to high-quality and timely care for preterm, LBW, and sick newborns is critical to improve outcomes. Support services for newborn referrals such as availability of transport and communications were poor in all study areas in rural and urban settings, resulting in refusal or delay of newborn referrals. Provision of ambulance services with trained staff for newborn referrals could improve health outcomes of preterm, LBW, and sick newborns presenting at higher level health facilities. Sensitization and training of health care providers on national referral protocols/guidelines, setting expectations for adherence, government investments in newborn referral systems, and standardizing initiating and receiving facility referral communication, are all urgently needed. Changes to the nationwide monitoring system to include performance of the referral system is crucial for accountability and improvement. Upgrading provision of newborn care at lower-level facilities will decrease the referral load at higher-level facilities.

## Data Availability

The qualitative data, individual stories and narratives have been collected in personal circumstances. Informants were assured that their contribution will remain confidential to the research project and will not be shared.

## Abbreviations

FGD: Focus group discussion
GAPPS: Global Alliance to Prevent Prematurity and Stillbirth
HEW: Health extension worker
IDI: In-depth interview
KII: Key informant interview
LBW: Low birth weight
MCH: Maternal and child health
PHCU: Primary health care unit
USAID: United States Agency for International Development
WHO: World Health Organization
